# Evaluating the Feasibility of ChatGPT in Healthcare: An Analysis of Multiple Clinical and Research Scenarios

**DOI:** 10.1007/s10916-023-01925-4

**Published:** 2023-03-04

**Authors:** Marco Cascella, Jonathan Montomoli, Valentina Bellini, Elena Bignami

**Affiliations:** 1 Department of Anesthesia and Critical Care, Istituto Nazionale Tumori - IRCCS, Fondazione Pascale, Via Mariano Semmola, 53, 80131 Naples, Italy; 2 Department of Anesthesia and Intensive Care, Infermi Hospital, AUSL Romagna, Viale Settembrini 2, 47923 Rimini, Italy; 3 Anesthesiology, Critical Care and Pain Medicine Division, Department of Medicine and Surgery, University of Parma, Viale Gramsci 14, 43126 Parma, Italy

**Keywords:** Artificial intelligence, ChatGPT, Medicine, Clinical resaerch

## Abstract

This paper aims to highlight the potential applications and limits of a large language model (LLM) in healthcare. ChatGPT is a recently developed LLM that was trained on a massive dataset of text for dialogue with users. Although AI-based language models like ChatGPT have demonstrated impressive capabilities, it is uncertain how well they will perform in real-world scenarios, particularly in fields such as medicine where high-level and complex thinking is necessary. Furthermore, while the use of ChatGPT in writing scientific articles and other scientific outputs may have potential benefits, important ethical concerns must also be addressed. Consequently, we investigated the feasibility of ChatGPT in clinical and research scenarios: (1) support of the clinical practice, (2) scientific production, (3) misuse in medicine and research, and (4) reasoning about public health topics. Results indicated that it is important to recognize and promote education on the appropriate use and potential pitfalls of AI-based LLMs in medicine.

## Introduction

Large Language Models (LLMs) are a type of Artificial Intelligence (AI) that are designed to mimic human language processing abilities. They use deep learning techniques, such as neural networks, and are trained on vast amounts of text data from various sources, including books, articles, websites, and more. Notably, extensive training enables LLMs to generate highly coherent and realistic text. LLMs analyze patterns and connections within the data they were trained on and use that knowledge to predict what words or phrases are likely to appear next in a specific context. This capability to comprehend and generate language is beneficial in various fields of natural language processing (NLP) such as machine translation and text generation.

Generative pre-training transformer (GPT) is a type of LLM model released by OpenAI (San Francisco, California), in 2018. It was trained using a variant of the transformer architecture on a dataset of 40GB of text and had a model size of 1.5B parameters [1]. Released in 2020, GPT-3 was trained on a massive dataset of text (570GB with a model size of 175B parameters). ChatGPT is the last variant of GPT-3, developed for dialogue with users [2].

Given its potential, the tool was immediately extensively tested. In a manuscript currently available as a preprint, ChatGPT passed the three exams of the United States Medical Licensing Exam (USMLE) [3]. Another study found that GPT-3.5 (Codex and InstructGPT) can perform at a human level on various datasets including USMLE (60.2%), MedMCQA (57.5%), and PubMedQA (78.2%) [4]. Despite the impressive outputs often produced by ChatGPT, it is unclear how well it will perform in the context of difficult real-world questions and scenarios, especially in fields such as medicine where high and complex mental loads are required [5]. Additionally, while the use of the chatbot in writing scientific articles may be useful, important ethical concerns arise [6].

On these premises, we used the publicly available webpage at https://chat.openai.com/chat to conduct a brief investigation for evaluating the potential use of ChatGPT in four clinical and research scenarios: (1) support of clinical practice, (2) scientific production, (3) misuse in medicine and research, and (4) reasoning about public health topics.

## ChatGPT for Supporting Clinical Practice

We started asking ChatGPT to compose a medical note for a patient admitted to the intensive care unit (ICU) after providing information regarding ongoing treatments, laboratory samples, blood gas analysis parameters, as well as respiratory and hemodynamic parameters, in a random order. After requesting a structured note, ChatGPT was able to correctly categorize most of the parameters into the appropriate sections, even when they were presented only as abbreviations and without any information about their meanings.

ChatGPT also showed an impressive ability to learn from its own mistakes and correctly assigned the right section to the previously misplaced parameters just by asking if that parameter was placed in the right section and without any other prompt. Notably, the major limitation was related to addressing causal relations among conditions such as acute respiratory distress syndrome (ARDS) and septic shock. It should be noted that while it was acknowledged that the sources of information may not be current or comprehensive enough to establish accurate causal connections. Additionally, ChatGPT was not designed for answering medical questions and, therefore, it lacks the medical expertise and context needed to fully understand the complex relationships between different conditions and treatments. Besides, ChatGPT demonstrated the ability to provide meaningful suggestions for further treatments based on the provided information, although at times the information provided was general. The best performance of ChatGPT was related to his ability to summarize information, although sometimes imprecise, using technical language for communication among clinics as well as plain language for communication with patients and their families.

## Scientific Writing

Moving towards potential applications of conversational AI-based tools in medical research, we evaluated chatGPT’s ability to understand and summarize information and draw conclusions based on the text from the Background, Methods, and Results sections of an abstract. To ensure that the provided information was not already known by the chatbot, whose current knowledge base is current up until 2021, we selected 5 papers published on the NEJM in the last months of 2022 [7–11]. Then, we wrote the following prompt “Based on the Background, Methods, and Results provided below, write the Conclusions of an abstract for the NEJM. The conclusions cannot be longer than 40 words”. Original and GPT-created conclusions are reported in Table 1. Overall, GPT was able to correctly indicate the setting and summarize the results of the primary outcome of the study. It was more likely to highlight secondary findings while the constraint of the text length was not strictly followed in favor of a meaningful message.

**Table 1:** The original abstract conclusions compared with the conclusions provided by GPT based on the Background, Methods, and Results provided from the abstract. The prompt given to GPT was “Based on the Background, Methods, and Results provided below, write the Conclusions of an abstract for the NEJM. The conclusions cannot be longer than 40 words.”

Article Title	Topic and design	Original abstract conclusions	Abstract conclusions provided by chatGPT	Ref.
Haloperidol for the Treatment of Delirium in ICU Patients	Multicenter, blinded, placebo-controlled trial on the treatment of ICU delirium	Among patients in the ICU with delirium, treatment with haloperidol did not lead to a significantly greater number of days alive and out of the hospital at 90 days than placebo.	Haloperidol showed no significant improvement in number of days alive and out of hospital at 90 days compared to placebo in adult ICU patients with delirium but was linked to lower mortality. Serious adverse reactions occurred at similar rates in both treatment groups. (43 words)	[7]
Defibrillation Strategies for Refractory Ventricular Fibrillation	Cluster-randomized trial with crossover on defibrillation approaches for refractory ventricular fibrillation	Among patients with refractory ventricular fibrillation, survival to hospital discharge occurred more frequently among those who received DSED or VC defibrillation than among those who received standard defibrillation.	DSED and VC defibrillation improve survival and neurologic outcomes in adult patients with refractory ventricular fibrillation during out-of-hospital cardiac arrest compared to standard defibrillation. DSED is more effective than VC defibrillation. (31 words)	[8]
Deferiprone in Parkinson’s Disease	Multicenter, phase 2, randomized, double-blind on the treatment of Parkinson’s disease	In participants with early Parkinson’s disease who had never received levodopa and in whom treatment with dopaminergic medications was not planned, deferiprone was associated with worse scores in measures of parkinsonism than those with placebo over a period of 36 weeks.	Deferiprone was associated with worse scores on measures of parkinsonism and decreased nigrostriatal iron content compared to placebo in participants with early Parkinson’s disease who had never received levodopa. However, the main serious adverse events with deferiprone were agranulocytosis and neutropenia. (41 words)	[9]
Prostate Cancer Screening with PSA and MRI Followed by Targeted Biopsy Only	Multicenter trial on screening for prostate cancer	The avoidance of systematic biopsy in favor of MRI-directed targeted biopsy for screening and early detection in persons with elevated PSA levels reduced the risk of overdiagnosis by half at the cost of delaying detection of intermediate-risk tumors in a small proportion of patients.	In a population-based screening study of 50–60 year old men, MRI-targeted biopsy was associated with a lower incidence of clinically insignificant prostate cancer compared to systematic biopsy. The relative risk of clinically significant prostate cancer was similar in both groups. Serious adverse events were rare in both groups. (48 words)	[10]
Trial of Anti-BDCA2 Antibody Litifilimab for Systemic Lupus Erythematosus	Multicenter trial on the treatment of Systemic Lupus Erythematosus	In a phase 2 trial involving participants with SLE, litifilimab was associated with a greater reduction from baseline in the number of swollen and tender joints than placebo over a period of 24 weeks. Longer and larger trials are required to determine the safety and efficacy of litifilimab for the treatment of SLE.	In a phase 2 trial of patients with SLE, 450 mg of subcutaneous litifilimab was associated with a decrease in the total number of active joints compared to placebo at week 24. Most secondary endpoints did not support the results of the primary endpoint analysis. Receipt of litifilimab was associated with adverse events, including herpes zoster and herpes keratitis. (59 words)	[11]

## Possible Misuse of GPT in Medicine and Research

We examined various applications that could result in both intentional and unintentional misuse. We also asked ChatGPT to suggest possible situations of misuse. In Table 2, we reported some of the suggestions provided by ChatGPT. Based on the responses, we assessed the technical feasibility. Although all the proposed settings of fraudulent use of ChatGPT are not exclusively of ChatGPT, what is impressive is the effective acceleration in the creation of fake evidence and materials with a high level of plausibility.

**Table 2:** Examples of possible misuse of GPT

Possible misuse	Request to ChatGPT	Feasibility (yes/no)
Using ChatGPT to fabricate research data or results to meet funding or publication requirements.	Provide codes in different languages (R software, STATA, SAS, Python) in order to create a dataframe with a certain mortality and variable distribution	Yes Assessor(s): 2 computer scientists (100% agreement)
Using the model to make diagnoses or treatment recommendations without proper validation or oversight.	Provide a diagnosis from medical history, clinical symptoms, and laboratory tests	Yes Assessor(s): 4 clinicians (100% agreement)
Generating fake news or misinformation.	Write 2 paragraphs to support the hypothesis of the laboratory origin of Sars-Cov-2 and natural origin, respectively	Yes Assessor(s): 4 clinicians (100% agreement)
Using ChatGPT to plagiarize or use someone else’s work as your own.	Generate a research paper by providing it with various scientific articles on a specific topic removing all the citations	Yes Assessor(s): 4 clinicians (100% agreement)
Using ChatGPT to generate data analysis that is not aligned with the actual data collected or not in line with the user’s stated purpose.	Generate a report on customer satisfaction, providing the model with a major prevalence of the responses of customers who had positive experiences	Yes Assessor(s): 4 clinicians (100% agreement)

Concerning the possible misuses proposed by ChatGPT, we also provided as a prompt a fictive dataframe in .csv format and asked to write the whole structured abstract for a scientific journal. Although the absence of a prompt with no information regarding the study (or the study aim), the first output was correctly structured with a plausible setting considering the variable name, realistic results, and coherent conclusions. Despite the fact that the abstract appeared to be reliable after a few prompts, it is important to consider that ChatGPT is not capable of performing statistical analyses and, upon different simulations, we noticed that it does not constantly advise on its limitations if not expressively requested. Interestingly, ChatGPT is able to assist and provide hints regarding codes for statistical analysis in different languages and even simulate model outputs of different types of models that might seem plausible to a reader who is not familiar with performing statistical analyses.

From these results, it is evident that this revolution in scientific publishing must be proactively managed through important regulatory policies. For example, there is a need to establish the margins of plagiarism [12].

## Reasoning About Public Health Topics

We investigated ChatGPT’s comprehension of the concept of seniority. The chatbot demonstrated the ability to provide accurate definitions and distinguished between the sociological and cultural definition of “elderly” and the medical definition, which defines individuals over the age of 65 as elderly and often vulnerable. Moreover, it divided the population of older adults into different subgroups based on their age or level of seniority, identifying three categories: young-old (the 60s or early 70s), middle-old (mid-70s to mid-80s), and old-old (over 85 years old). This categorization aligns with definitions used in various studies [13]. The chatbot also mentioned an important concept in medicine, namely that “It is important to note that these categories are generalizations and that individual older adults may experience different health needs and challenges regardless of their age”.

Then, we asked ChatGPT if it was possible to objectively measure a person’s seniority from a biological perspective. The chatbot promptly provided a list of commonly studied methods, including dental and skeletal development, telomere length, DNA methylation, hormone levels, and cognitive functioning.

The next question we posed to GPT was more complex. We asked if it would be useful to examine patients’ objective age-related factors in the perioperative context. The chatbot gave us an affirmative answer and, upon request, provided clinical studies to support its response: “For example, a study published in the journal Anesthesiology in 2016 found that older patients with shorter telomeres … were more likely to experience post-operative complications… Other studies have examined the role of hormone levels, such as testosterone and estrogen, in the perioperative period. A study published in the Journal of Anesthesia in 2017 found that older men with lower testosterone levels were more likely to experience complications after major surgery, while a study published in the journal Anesthesiology in 2019 found that older women with lower estrogen levels were at increased risk of post-surgical cognitive decline”.

Finally, we tried to contextualize previous findings in clinical research by asking “What would it be interesting to study about the objectivity of age and perioperative period?”. The chat responded in an interesting manner, and it listed four possible research topics:

1. Identifying and validating additional objective age-related biomarkers.
2. Examining the impact of interventions on objective age-related biomarkers.
3. Investigating the potential role of objective age-related biomarkers in personalized medicine.
4. Evaluating the impact of objective age-related biomarkers on long-term outcomes.

According to this test, we have noticed that when discussing public health topics, the chatbot is able to provide accurate definitions and can even give examples of clinical studies. However, some of the responses may be stereotyped and the logical connections may depend on the user’s input.

In conclusion, NLP-based models could have great potential to accelerate science and improve scientific literacy by supporting various aspects of research. On a larger scale, they could be useful in exploring the literature and generating new research hypotheses. Additionally, these strategies can serve for handling complex data, as well as for extracting useful information from medical texts, such as electronic health records (EHRs), clinical notes, and research papers. Finally, they may facilitate the dissemination of scientific findings by translating complex research into more easily understandable language for the general public.

On the other hand, it is crucial for the scientific community to understand the limits and capabilities of ChatGPT. This entails determining the specific tasks and areas for which ChatGPT can be well-suited, as well as any potential challenges or limitations. The so-called “hallucination” phenomenon, for example, refers to the ability of ChatGPT to produce answers that sound believable but may be incorrect or nonsensical. Additionally, another great problem is that ChatGPT can reproduce biases present in the data it was trained on.

By establishing a clear understanding of ChatGPT’s abilities and limits, researchers and practitioners can utilize the technology effectively, while avoiding any unintended consequences. Furthermore, by identifying these boundaries, the community can also identify areas where further research and development are needed for improving the model’s performance and capabilities. To date, due to their significant limitations, many challenges arise for the applications of these instruments for both clinical aid and research purposes [14].
